# Early effects of exposure-based cognitive behaviour therapy on the neural correlates of anxiety

**DOI:** 10.1038/s41398-018-0277-5

**Published:** 2018-10-19

**Authors:** Andrea Reinecke, Kai V. Thilo, Alison Croft, Catherine J. Harmer

**Affiliations:** 1 Department of Psychiatry, University of Oxford, Oxford, UK; 2 Oxford Psychologists Ltd, Oxford, UK; 3 Oxford Cognitive Therapy Centre, Warneford Hospital, Oxford, UK

## Abstract

Exposure-based cognitive-behaviour therapy (CBT) for anxiety disorders is an effective intervention, but the brain mechanisms driving recovery are largely unknown. In this experimental medicine study, we investigated to what degree CBT affects neural markers of anxiety at an early stage of treatment, to identify dynamic mechanistic changes which might be crucial in the process of recovery as opposed to those seen following full treatment completion. In a randomised controlled trial, unmedicated patients with panic disorder either received four weekly sessions of exposure-based CBT (*N* = 14) or were allocated to a waiting group (*N* = 14). Symptom severity was measured before and after the intervention. During functional magnetic resonance imaging (fMRI), patients performed an emotion regulation task, either viewing negative images naturally, or intentionally down-regulating negative affect using previously taught strategies. Four-session CBT led to marked reductions in symptoms and 71% of patients reached recovery status (versus 7% in the control group). This intervention normalised brain hyperactivation previously seen in panic disorder, particularly in areas linked to threat monitoring, fear memory, and maladaptive emotion regulation, such as amygdala, dorsomedial and dorsolateral prefrontal cortex, and temporal gyrus. Our findings suggest that optimal treatment doses for panic disorder might be much lower than previously thought. Furthermore, this is the first study to show that neural markers of anxiety change very early during CBT, highlighting potential neural mechanisms that might drive clinical recovery. Such knowledge is important for the development of more compact combination treatments targeting these mechanisms more effectively. (Neural Effects of Cognitive-behaviour Therapy in Panic Disorder; clinicaltrials.gov; NCT03251235)

## Introduction

Exposure-based cognitive-behaviour therapy (CBT) for anxiety disorders—the human equivalent to fear extinction in animal models involving exposure to fear-provoking situations to dispute catastrophic expectations—is an effective first-line treatment^1^. However, its neural mechanisms of action remain largely unclear, even though a better understanding may help guide future treatment development. It is already well known that anxiety disorders are associated with a distinctive pattern of altered responsivity in a network of limbic and prefrontal-cortical brain regions. In particular, it has been demonstrated that during threat processing, anxiety patients show increased activation in amygdala, occipital gyrus and dorsal areas of anterior cingulate (dACC) and medial prefrontal cortex (dmPFC), brain regions associated with threat detection and monitoring^2–4^. In addition, anxiety is associated with increased activation in a range of ventral and lateral prefrontal-cortical regions known to be implicated in emotional control^5^. Most importantly, these patterns of hyperactivation in limbic and prefrontal areas have been shown to resolve with recovery after conventional long-term courses of CBT in phobia, OCD and social anxiety^6–10^, reinforcing the idea that such functional brain alterations play a key role in the pathogenesis of a disorder.

However, these early findings must also be interpreted with caution since they often include patients treated with medication which can also affect neural responses to emotional information, and the only study looking at neural effects of CBT in panic disorder lacked a no-treatment patient group to control for spontaneous remission effects^11^. Furthermore, it remains to be explored to what degree CBT affects neural markers of anxiety at an early stage of treatment. Such an approach would highlight the dynamic mechanistic changes which are important in the process of recovery as opposed to those seen following full treatment completion.

In this experimental medicine study, we investigated neural response to threat images while either maintaining negative affect or using taught strategies of reappraisal. We compared patients with panic disorder after brief CBT of only four weekly sessions to a patient waiting group. Our previous work had shown that compared to healthy volunteers, panic patients show increased activation in limbic and prefrontal areas during Maintain blocks, while they show no differences during Reappraisal blocks. Based on our previous work showing that a single session of CBT already significantly reduces vigilance for fearful faces^12^, we hypothesised that brief CBT would reduce limbic and prefrontal response to threat images.

## Methods and materials

### Participants

Formal sample size calculation was limited by the lack of previous evidence regarding the effect of very brief exposure therapy on neuroimaging outcomes. We estimated sample size based on our previous work looking at the effects of single-session CBT on attention bias (treated group *M* = 5/SD = 30, waiting group *M* = 39/SD = 38; *N* = 14 per group; *d* = 0.99^12^), suggesting sample sizes of 14 to achieve a power of 80% for a one-sided between-group test at an alpha-level of 0.05. We therefore aimed to test 14–16 participants per group in this study. Thirty-four patients with a current diagnosis of panic disorder with or without agoraphobia were recruited from the general public through newspaper adverts, flyers in GP and clinical psychologist practices, and posters in public places. In a parallel study design, patients were assigned to a CBT treatment group (TG) receiving immediate treatment versus a waiting group (WG) receiving treatment after 4 weeks of waiting, using blocked randomisation while stratifying for gender. The randomisation sequence was generated by a researcher not in direct contact with participants (CH), based on random number strings generated by Excel. In this trial, it was unfeasible for participants to remain blind to group allocation. We aimed to reduce bias by leaving therapists blind to randomisation, and by having an investigator not involved in treatment being responsible for enrolment and testing of patients on most outcome measures (AR). Two patients allocated to the TG and three patients allocated to the WG withdrew from participation before the intervention due to inability to commit to the time scale of the study, leaving 14 volunteers per group for analyses.

Diagnoses were assessed using the Structured Clinical Interview for DSM-IV Axis I Disorders^13^. Exclusion criteria were left-handedness, MRI contraindications, lifetime history of epilepsy, psychotic disorder, bipolar disorder, or substance abuse, antidepressant treatment during the last 6 months, previous CBT, primary diagnosis other than panic disorder. Occasional benzodiazepine or beta-blocker medication was not an exclusion criterion but patients refrained from these drugs 48 h before treatment and scanning sessions (benzodiazepine: 5 TG, 2 WG; beta-blocker: 0 TG, 1 WG, both *p* > .65). Groups were well matched in terms of age, gender, educational level, verbal intelligence (NART;^14^) (Table 1), and primary (panic disorder: 4 TG, 6 WG; panic disorder with agoraphobia: 10 TG, 8 WG; *χ*^*2*^ = 0.62, *df* = 1, *p* = 0.430) and comorbid diagnoses (social phobia: 2 TG, 1 WG; specific phobia: 2 TG, 3 WG; *χ*^*2*^ = 0.53, *df* = 2, *p* = 0.766). All study procedures took place at the University of Oxford Department of Psychiatry, and the Oxford Centre for Magnetic Resonance at the John Radcliffe Hospital, Oxford. Ethical approval was obtained from the South Central—Oxford A ethics committee. All participants gave written informed consent. In combination with additional data some of the WG data has previously been published^15^.

**Table 1:** Socioeconomic, mood and anxiety questionnaire scores in the two groups (*M* ± *SD*, independent-samples *t*-test/ *X*^*2*^-test *P*-scores, Cohen’s *d*)

	Treatment (*N* = 14)		Waiting (*N* = 14)		*P*	*d*
	*M*	*SD*	*M*	*SD*		
Sociodemographic data						
Age	34.8	14.6	37.2	11.1	.63	
Years of education	15.2	2.6	15.8	2.5	.56	
Verbal IQ (NART)	118.0	4.78	116.6	5.6	.48	
Baseline measurements						
CGI-S	4.5	0.8	4.7	0.6	.42	0.3
HADS–anxiety	14.4	4.2	13.4	3.7	.51	0.3
HADS depression	8.4	4.6	9.1	3.7	.62	0.2
BSQ	3.4	0.4	3.4	0.8	.75	0.0
ACQ	2.5	0.7	2.3	0.6	.54	0.3
After 4-week CBT/waiting						
HADS–anxiety	6.1	4.0	12.9	3.5	<.001	1.8
HADS depression	2.6	2.6	8.6	3.9	<.001	1.8
BSQ	1.8	0.8	3.3	0.9	<.001	1.8
ACQ	1.5	0.5	2.4	0.7	.001	1.5
Negative affect during scan						
Reappraisal	1.6	0.8	1.8	0.8	.74	0.3
Maintain	2.7	0.9	2.6	0.9	.43	0.1

*NART* national adult reading test, *CGI-S* clinical global impression–severity, *HADS* hospital anxiety and depression scale, *BSQ* body sensations questionnaire, *ACQ* agoraphobic cognitions questionnaire

### Clinical symptoms

Before treatment, the clinician-administered Clinical Global Impression Scale-Severity (CGI-S) rating was applied (1 = not at all ill to 7 = among the most extremely ill patients). After treatment, the CGI-Improvement (CGI-I) was used (1 = very much improved to 7 = very much worse)^16^. At baseline and after 4-week treatment (TG) or waiting (WG), participants completed the (i) Hospital Anxiety and Depression Scale (HADS, each subscale ranging from 0–21;^17^), (ii) Agoraphobic Cognitions Questionnaire (ACQ, range 1–5,^18^), and (iii) Body Sensations Questionnaire assessing fear of physical sensations (BSQ, range 1–5;^18^).

### fMRI task design

Patients were brain scanned after 4-week CBT versus waiting using an emotion regulation task that has previously been shown to differentiate between patients with panic disorder and healthy volunteers^5^. Forty negatively valenced coloured IAPS images^19^ mainly showing panic-related catastrophic expectations (e.g., intensive care scenes, funerals) were presented in 8 blocks of 5 images (5 s each image), alternating with grey fixation baseline blocks (30 s). For half of the blocks, participants were instructed to naturally experience the emotional state evoked by the images, without attempting to regulate or alter it (Maintain blocks). For Reappraisal blocks, they were instructed to down-regulate the provoked negative affect by using previously demonstrated strategies of cognitive reappraisal (e.g., reframing, rationalising). At the end of each picture block, participants indicated the intensity of negative affect experienced throughout the block using a keypad (1 = neutral, 4 = negative).

### Image acquisition

3T Siemens Sonata functional imaging data were analysed using FEAT 6.0, part of FSL (FMRIB Software Library; www.fmrib.ox.ac.ul/fsl) with Z > 2.3 and *p* < .05. T_2_*-weighted functional data were acquired for a whole-brain field-of-view (64 × 64 × 40 matrix, 45 slices, voxel resolution 3 mm^3^, gap 1.5 mm, repetition time (TR) = 3000 ms, echo time (TE) = 30 ms, flip angle = 90°). Field maps were acquired using a dual echo 2D gradient echo sequence with echos at 5.19 and 7.65 ms, and a repetition time of 500 ms. High-resolution T1-weighted images were acquired for subject alignment, using an MPRAGE sequence with the following parameters: 174 × 192 × 192 matrix, voxel resolution 1 mm^3^, TR = 2040 ms, TE = 4.7 ms, inversion time (TI) = 900 ms.

### Image analysis

Pre-processing included motion correction^20^, non-brain removal^21^, spatial smoothing (Gaussian kernel FWHM = 5.0 mm), grand-mean intensity normalisation of the entire 4D dataset by a single multiplicative factor, registration of the functional space template to the anatomical space and the MNI 152 space, highpass temporal filtering (Gaussian-weighted least-squares straight line fitting, with sigma = 50.0 s), fieldmap correction. At the first-level, data were analysed using a general linear model approach with local autocorrelation correction^22^. Two regressors of interest (Maintain, Reappraisal) and two regressors of no interest (instruction/ rating periods) were included. Fixation blocks were the implicit baseline reference. Contrast images were calculated for picture blocks, Maintain blocks, Reappraisal blocks, Maintain versus Reappraisal, Reappraisal versus Maintain. Individual activation maps were then entered into the group level, using a mixed-effects whole-brain analysis^23^.

Based on our previous work identifying the amygdala as hyperactive in Maintain versus Reappraisal blocks in patients with panic disorder compared to healthy volunteers^5^, we ran group comparisons in a bilateral amygdala region of interest (ROI), including 10 mm radius spherical masks around a previously published peak voxel of a left amygdala region (-14/-6/-8) and its right-hemisphere counterpart (14/-6/-8)^24^. Significant whole-brain or ROI interactions were explored by extracting percent blood oxygenation level-dependent (BOLD) signal changes and entering these into a Group × Task (Maintain, Reappraisal) ANOVA. They were further explored running two-tailed Pearson’s correlation analyses for percent signal change and panic severity (mean of the scores achieved on ACQ and BSQ).

In an exploratory one-way ANOVA, we also compared mean amygdala signal measured in panic patients after 4-session CBT or 4-week waiting to that seen in healthy controls in a previous study using this task (*N* = 18, Maintain: 0.14 ± 0.17, Reappraise: 0.18 ± 0.15)^5^, to be able to estimate the clinical relevance of any effects of 4-session CBT on amygdala response.

#### Connectivity analysis

As we have previously identified altered functional connectivity of left and right amygdalae in panic patients compared to controls using this task^5^, we ran identical analyses here. At the first level, we extracted for each participant a deconvolved time series for a) the functional picture blocks versus baseline cluster identified within the anatomical right amygdala using small volume correction and b) the functional cluster within the anatomical left amygdala. These time courses were then entered into two FSL psychophysical interaction (PPI) analyses (right amygdala versus left amygdala cluster as seed region), along with the two psychological regressors (Maintain, Reappraisal, picture blocks), the two PPI regressors (Maintain × timeseries, Reappraisal × timeseries) and the regressors of no interest (instructions, ratings). Individual contrast images were then entered into the group level, using a mixed-effects whole-brain analysis.

#### Cognitive-behavioural treatment

Therapists were four psychology graduates trained in delivering protocol-driven CBT through an Oxford University outreach service for panic disorder, with data showing treatment quality similar to that of clinical psychologists^25^. Training and supervision were provided by an experienced clinical psychologist (AC), and treatment adherence was monitored via review of written session protocols during supervision. Treatment was a condensed version of routine clinical care intervention and involved four weekly sessions of exposure-based CBT, based on the well-established cognitive-behavioural theory of panic^26^. This approach assumes that anxiety disorders develop as a consequence of neutral physical sensations (e.g., increased heart rate) being misperceived as threatening (e.g., I am having a heart attack), and the use of maladaptive safety strategies (e.g., taking propranolol tablet, leaving situation) preventing corrective experiences (e.g., I will not die of a heart attack even if I am not leaving the situation.).

Treatment involved cognitive and behavioural components, with the following key ingredients: (i) idiosyncratic assessment to establish an individual hierarchy of feared situations, catastrophic expectations, and safety strategies used to prevent the anticipated catastrophe, (ii) cognitive preparation: explanation of individually relevant learning mechanism underlying the development and treatment of anxiety, especially the role of safety strategies, (iii) repeated exposure to fear- provoking situations and bodily sensations while dropping all safety strategies, to test out catastrophic expectations and break through stimulus-driven response cycles, (iv) cognitive debriefing to discuss the patient’s experience in a threatening situation without safety strategies, and to consolidate this behaviour. In comparison to standard longer-term CBT where exposure often involves gentle step-by-step confrontation with situations listed in the patient’s individual fear hierarchy, therapists in this study encouraged patients to exposure to situations higher up in the hierarchy right from the start.

### Statistical analysis of behavioural data

Differences in negative affect ratings were analysed running Group × Task mixed-model analyses of variance (ANOVA) and post-hoc two-tailed *t*-tests, using SPSS 20 (*ɑ* = 0.05). Group differences in symptom questionnaire scores were analysed running group (treatment, waiting) × time (baseline, post treatment/waiting) ANCOVA entering baseline symptom severity as a covariate, and follow-up two-tailed independent-samples and paired *t*-tests. In line with common standards in CBT for anxiety research^27^, ‘response’ was defined as having reached a CGI-I score of 1 or 2, and ‘recovery’ was assumed when a CGI-I score of 1 or 2 was accompanied by healthy-range BSQ and ACQ scores^18^.

## Results

### Affect ratings and behavioural data

At baseline, the groups showed similar symptom severity on all measures (Table 1), and panic severity scores were comparable to those seen in primary care settings^28^. During 4-week CBT, there was a significant reduction of trait anxiety and depression (HADS), fear of physical sensations (BSQ), and agoraphobic cognitions (ACQ) in treated patients (all F *>* 17.2, *df* = 1/27, all *p* < 0.001, all *d* > 2.09; all *t* *>* 4.8, *df* = 13, all *p* < 0.001), leading to significant group differences compared to the waiting group on all these measures at retest (all *p* < 0.002, all *d* > 1.5). All patients in the treatment group reached CGI-I ratings of 1 or 2 (*M* = 1.5, *SD* = 0.5). Although before treatment all patients in both groups reported a clinically significant severity of fear of physical sensations (BSQ) and agoraphobic cognitions (ACQ), 71% of treated patients fulfilled criteria for recovery at the end of treatment, with both BSQ and ACQ scores falling within the range reported for healthy control subjects (cut-offs ACQ: 2.06, BSQ: 2.39)^18,29^. In comparison, this was true for only one waiting group patient (*χ*^*2*^ = 19.3, *df* = 1, *p* < 0.001). Pre-post effect sizes (Cohen’s *d*) in the treatment group were large for both BSQ (*d* = 2.5) and ACQ (*d* = 1.6). Negative affect ratings were lower in Reappraisal versus Maintain blocks in both groups, without any between-group differences (Task *F* *=* 16.1, *df* = 1/24, *p* = 0.001; Group/ Group × Task both *p* > 0.420).

### BOLD fMRI

#### Whole-brain analysis

##### Main effect of Task (Reappraise versus Maintain, across groups)

In line with our own and other groups’ previous work^5,24,30^, Reappraisal was associated with increased activation in bilateral areas of dorsal ACC, dmPFC, dlPFC, vlPFC, orbitofrontal cortex, and insula (18201 voxels, MNI −4,24,44, *Z* = 5.15, *p* < 0.001), bilateral cerebellum extending into occipital fusiform gyrus (left: 406 voxels, MNI −54,−60,−32, *Z* = 3.39, *p* = 0.0084; right: 1557 voxels, MNI 32,−62,−52, *Z* = 4.53, *p* < 0.001), and bilateral angular gyrus (left: 355 voxels, MNI −48,−40,30, *Z* = 3.86, *p* = 0.019; right: 507 voxels, MNI 54,−48,48, *Z* = 4.07, *p* = 0.0018).

##### Main effect of Group (Picture blocks versus baseline)

**Fig. 1:**
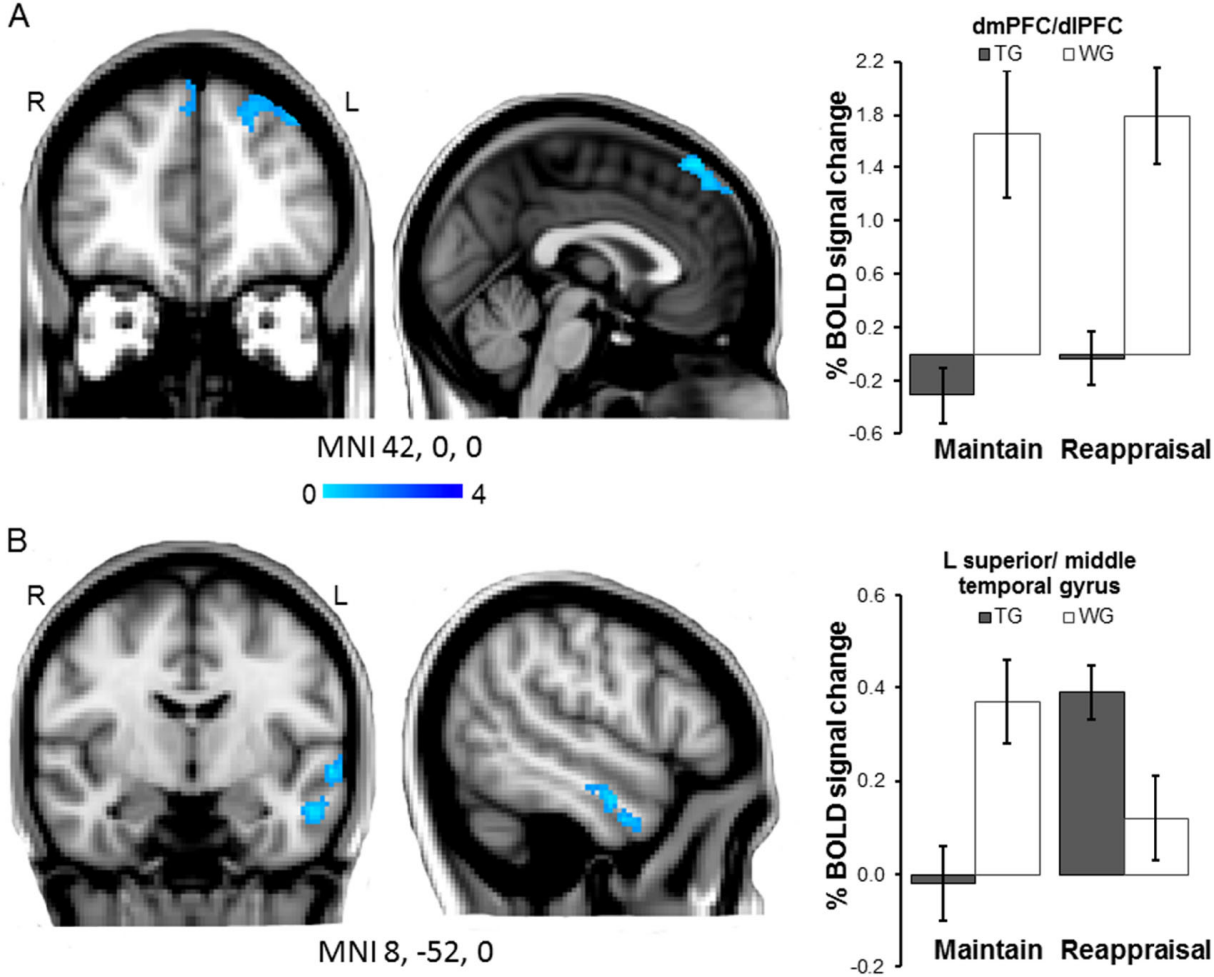
Whole-brain analysis. **a** Main effect of Group: Compared to waiting list patients (WG), treated patients (TG) show reduced activation in left and right dmPFC and left dlPFC during picture blocks versus fixation baseline blocks (Maintain/Reappraisal both Maintain *p* < .001). **b** Group × task interaction: Maintaining negative affect versus Reappraisal was associated with attenuated signal response in treated compared to untreated patients in the left anterior superior-middle temporal gyrus (Maintain *p* < .01; Reappraisal *p* < .05). Images thresholded at *Z* > 2.3, *P* < 0.05, corrected. Note*:* Error bars show SEM

Compared to the waiting group, treated patients showed significantly reduced activation in bilateral dmPFC and left dlPFC during the picture blocks versus the fixation screen baseline (540 voxels, MNI −2,36,60, *Z* = 4.59, *p* = 0.0033; main sub-regions within this cluster: MNI −36,38,42, *Z* = 3.52, MNI 2,46,50, *Z* = 3.46) (Fig. 1a). Percent signal change in this cluster was not correlated with panic symptom severity in any of the groups (all *p* > 0.130).

##### Group × task interaction (Maintain versus Reappraisal)

There was a significant Group × Task interaction in a cluster in the left middle and superior temporal gyrus (356 voxels, MNI −58,−4,−10, *Z* = 4.53, *p* = 0.019; main sub-region within this cluster: MNI 54,−6,−24, *Z* = 3.60) (Fig. 1b). Post-hoc analyses on BOLD signal change extracted from this cluster indicated that this interaction was driven by converse group differences in activation during Maintain and Reappraisal Blocks (ANOVA Task × Group *F* *=* 39.4, *df* = 1/26, *p* < 0.001, *d* = 2.45), with treated patients showing significantly reduced activation compared to the waiting group in Maintain blocks (*t* = 3.2, *df* = 26, *p* = 0.003, *d* = 1.90) and relatively increased activation in Reappraisal blocks (*t* = 2.4, *df* = 26, *p* = 0.020, *d* = 0.93). Activation in this cluster during Maintain minus Reappraisal blocks was not correlated with symptom severity in any of the groups (all *p* > 0.120).

#### Amygdala ROI analysis

**Fig. 2:**
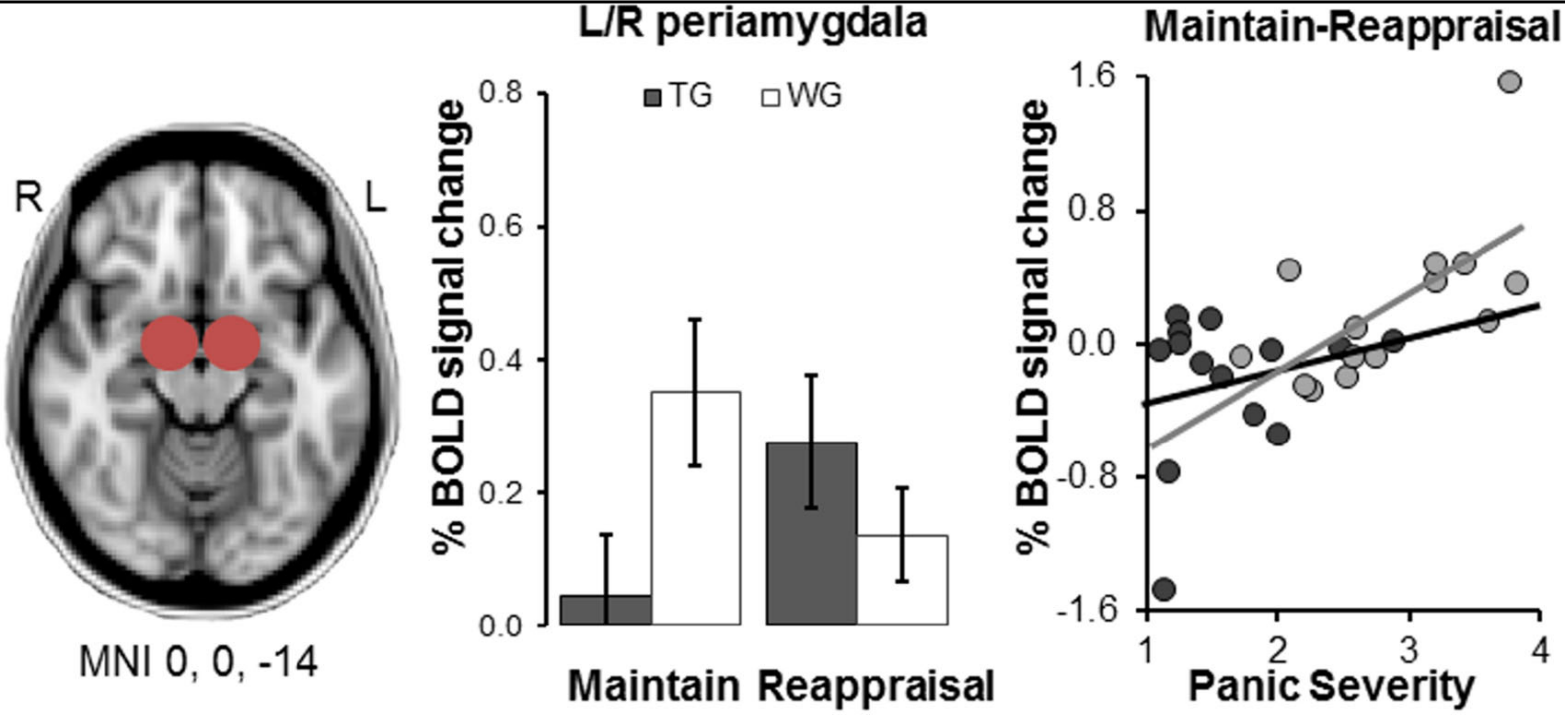
Region of interest analyses in left and right amygdala clusters. Compared to waiting list patients (WG), treated patients (TG) show reduced activation during Maintain blocks (p < .05). BOLD % signal change during Maintain minus Reappraisal blocks across both amygdalae was positively correlated with panic severity in untreated (WG, p = .01) but not treated patients (TG). Note: Error bars show SEM. Panic severity is calculated as the mean of the scores achieved on ACQ and BSQ

A Group × Task ANOVA for the BOLD percent signal change extracted from the bilateral amygdala ROI (Maintain: TG .05 ± .32, WG .39 ± .42; Reappraise: TG .22 ± .42, WG .15 ± .26) revealed a significant interaction (*F* = 6.5, *df* = 1/26, *p* < 0.020 *d* = 1.00). This effect was driven by treated patients showing reduced amygdala activation compared to the waiting group in Maintain blocks (*p* < 0.040, *d* = 0.83), but not in Reappraisal blocks (*p* = 0.290, *d* = 0.33). Increased Maintain minus Reappraisal percent signal change was associated with increased panic severity in the waiting group but not treated patients (WG: *r* = .63, *p* = 0.010; TG: *r* = .23, *p* = 0.440) (Fig. 2).

Comparing amygdala signal measured in this study to that previously seen in healthy controls using the same task, untreated (*p* = 0.050, *d* = 0.68) but not treated patients (*p* = 0.290, *d* = 0.37) showed significantly higher amygdala signal during Maintain than healthy volunteers *F* *=* 3.7, *df* = 2/45, *p* = 0.034, *d* = 0.838).

### fMRI connectivity analysis

**Fig. 3:**
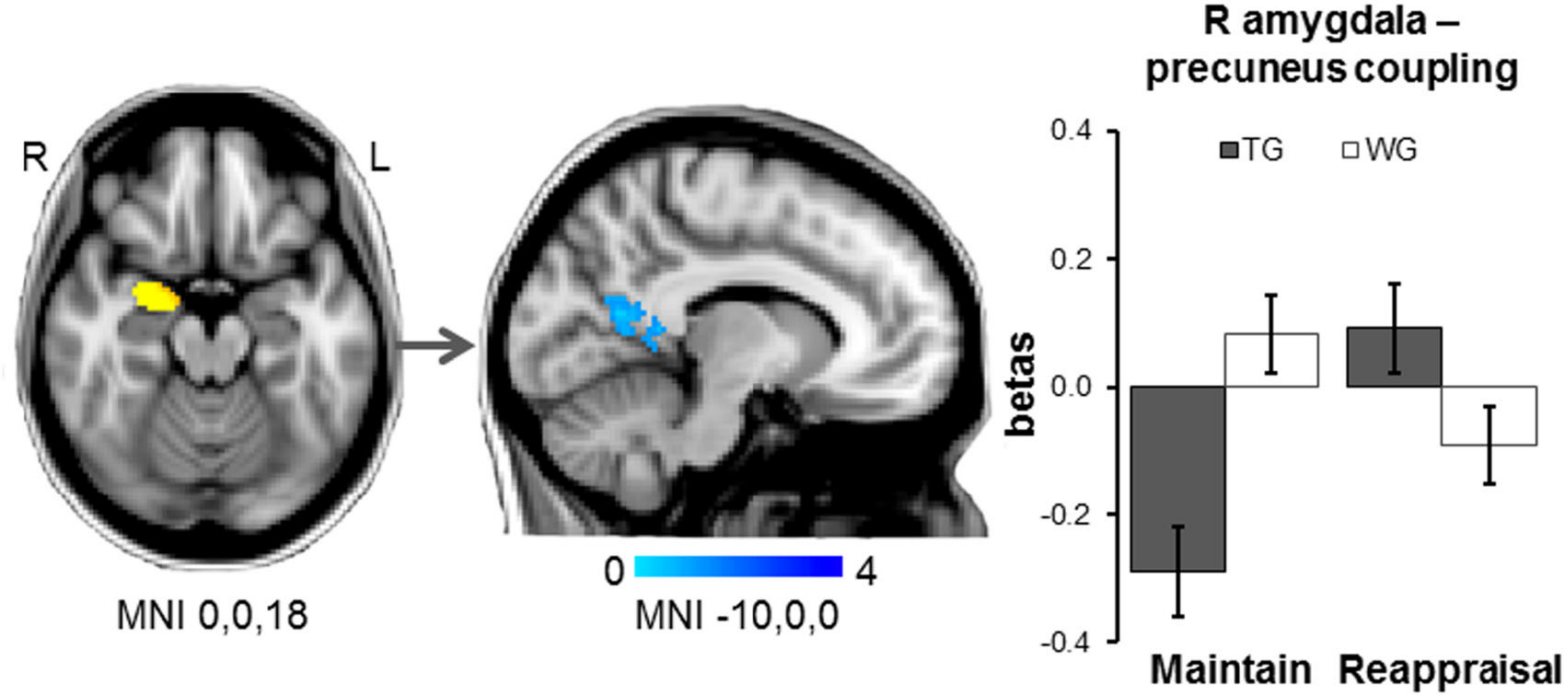
Whole-brain psychophysiological interaction analyses. Using a right amygdala functional cluster (picture blocks versus baseline, acrossgroups) as the seed region, waiting list patients (WG) compared to treated patients (TG) showed reduced connectivity of the right amygdala with aleft precuneus/ posterior cingulate cortex cluster during Maintain blocks versus Reappraisal (Maintain: independent-samples t-test p thresholded at Z > 2.3, P < 0.05, corrected. Note: Error bars show SEM

While untreated patients showed a positive correlation of activity in the right amygdala during *Maintain blocks (versus Reappraisal)* with activity in a left precuneus–ventral posterior cingulate cluster (275 voxels, MNI −14,−58,8, *Z* = 3.70), this association was significantly reduced and reversed in treated patients. No significant patterns of connectivity were identified for the left amygdala seed (small volume correction; picture blocks versus baseline, across groups; right amygdala peak: 24,−4,−18; *Z* = 5.90; left amygdala peak: −18,−4,−16; *Z* = 6.53) (Fig. 3). Maintain minus Reappraisal magnitudes of right amygdala–left precuneus coupling were not correlated with symptom severity in either group (all *p* > 0.320).

## Discussion

This study explored the effect of four sessions of CBT for panic disorder on the neural correlates of fear reactivity. We found that a brief treatment altered activation in limbic, paralimbic and prefrontal brain areas in patients with panic disorder. These findings are consistent with studies showing a normalisation of neural activity following standard longer-term CBT in a range of anxiety disorders^7–9^ and suggest that these changes can be observed very early in treatment and relative to a control group. Our results also show that this brief treatment led to significant clinical improvement, with 71% of treated patients reaching recovery status. This level of clinical response after only four sessions of CBT is comparable to recovery rates after standard longer-term CBT for panic disorder, as estimated by a recent meta-analysis^31^, and might be attributed to our CBT protocol rapidly tackling exposure situations higher up in the fear hierarchy of the patient

### Early neurobiological effects of CBT

Four sessions of CBT reduced activation in bilateral dmPFC and left dlPFC in response to emotional pictures overall, and reduced activation in amygdala and left middle-superior temporal gyrus during uninstructed emotion regulation (Maintain versus Reappraisal). Exploratory analyses revealed that the amygdala signal in the CBT group was comparable to that of healthy controls, suggesting that this brief treatment significantly attenuates aberrant activity in this key area of threat detection. Increased activation in all these areas of threat detection and emotion regulation had been identified as relevant in anxiety in a previous study using an identical fMRI task in patients versus healthy volunteers^5^. Our current findings corroborate that this pattern of hyperactivation is targeted very early on during psychological treatment.

These findings are in line with recent neurobiological accounts of anxiety, linking a disorder to increased activation in a network of the brain including occipital, limbic, and dorsal-medial PFC regions relevant for threat detection and monitoring^32–34^. These models also highlight that anxiety is often associated with increased activation in lateral and ventral PFC areas of inhibitory control, perhaps reflecting maladaptive, avoidant regulation attempts and safety strategies typically seen in these disorders. Our finding of a reduction of activation in all these regions may indicate that brief CBT leads to emotional processing being less biased towards threatening stimuli, and that previous threat stimuli may have ceased to automatically signal danger and trigger fear responses. Such findings challenge common assumptions that anxiety may be associated with decreased activation in prefrontal-cortical control areas, and that improved emotion regulation throughout the course of CBT would lead to an increase in activation^35^. Instead, they support the hypothesis that overactivity may reflect ineffective regulation, which becomes more efficient following CBT.

The present study also indicates a key role of the left anterior temporal gyrus in early response to CBT. Even though rarely explicitly discussed, this area is often listed as being overly activated in anxiety patients during fear processing^36–38^ and as being sensitive to treatment^39^. Anatomically, the anterior temporal lobe is highly interconnected with limbic, prefrontal, and sensory-motor areas of the brain, therefore being ascribed a gateway role in linking information from different modalities to form and retrieve emotional memory representations^40–42^.

Our results also demonstrate that brief CBT significantly alters patterns of neural connectivity. Treated patients showed reduced positive connectivity between a right amygdala and a left precuneus–posterior cingulate cortex cluster, regions thought to be key nodes of the default mode network^43^. While deactivation in this network is associated with an adaptive allocation of attention towards task-relevant external events, increased activation has been associated with interfering internal processes such as internally-directed attention, arousal, vigilance during the anticipation of unknown stimuli, and retrieval of emotional, personally-relevant memories^44^. In line with these observations, increased activity in these regions has been reported for a range of anxiety disorders^45^. Our finding of decreased connectivity between these regions following brief CBT highlights actions in default mode processing as a potential target for treatment in panic disorder.

### Implications

This study demonstrates that neural markers of anxiety shift very early during CBT, emphasising the possibility that these rapid changes at the brain level may drive clinical effects of treatment. This hypothesis is in line with our previous work showing that a single session of CBT leads to fast changes in behavioural markers of threat vigilance, which predict symptom recovery over a 1-month follow-up period^12^.

While the same relationship remains to be formally demonstrated for neural changes seen in the present study, previous research suggests that the brain areas targeted by this brief CBT treatment, such as amygdala or dorsomedial PFC, are the neural correlates of threat vigilance^3,4^. It may therefore be possible that these neural changes represent a more fundamental, underlying mechanism of CBT action. In interaction with environmental factors, dampened reactivity of brain areas associated with threat detection and monitoring may help accumulate additional positive exposure experiences, which in turn translate into therapeutic effects. Such a hypothesis is consistent with recent ideas about the mechanisms of anxiolytic and antidepressant drug treatment^46^ and highlight the possibility that pharmacological and psychological treatment may work more similarly than previously thought.

While these results are promising, there are several limitations. First, our results allow no final conclusions as to whether the observed changes in threat processing in the brain throughout brief CBT are driving or are a consequence of symptom improvement. Future research using even smaller CBT ‘doses’ to be able to observe neural changes prior to symptom changes are therefore required. One might also assume that the study design involving an MRI scan might lead to an artificial pre-selection of patients with less severe panic disorder with or without agoraphobia. However, our samples show panic severity scores comparable to those seen in primary care settings^28^. Another limitation of this study relates to the fact that the clinical assessor, while not involved in therapy, was not blind to group allocation of patients. Also, while recovery rates of 71% seen after this brief CBT protocol are very encouraging and suggest that standard treatments might be developed into more economic, condensed formats, this study allows only limited conclusions regarding the stability of these effects, and future work will have to establish that relapse rates are not inferior to those seen after conventional treatment. Further limitations of this study are the small sample size and absence of baseline fMRI measures. These early effects of CBT observed here within fear networks should be replicated in larger samples with baseline assessment to test for prediction of therapeutic response.

Taken together, this is the first study to show that neural markers of anxiety normalise very early during CBT, highlighting potential neural mechanisms that might drive clinical recovery. Furthermore, our findings suggest that optimal treatment doses might be much lower than previously thought, bringing forward an ultra-brief exposure treatment that leads to recovery rates similar to those seen after standard longer-term CBT. Such knowledge is important for the development of future treatments Identifying add-on CBT components such as pharmacological compounds that boost these early changes in threat processing further may have potential to ultimately develop brief CBT into standard-of-care treatment.

## Electronic supplementary material

Supplementary Figure 1
