# Supplementary material for: Early effects of exposure-based cognitive behaviour therapy on the neural correlates of anxiety
Source: Transl Psychiatry. 2018 Oct 19;8:225. doi: 10.1038/s41398-018-0277-5 (PMC6195621; doi:10.1038/s41398-018-0277-5)
Supplement: Supplementary file 1 — Supplementary Figure 1 [file 41398_2018_277_MOESM1_ESM.docx]

**Enrolment**

**Step 1 eligibility assessment: n=210**

Online screening questionnaire PDSS

**n=120**

**Excluded:**

*PDSS score < 4

**Step 2 eligibility assessment: n=90**

Email pre-screening

**n=48**

n=18

n=18

**Excluded:**

*medication

*other anxiety/ depression

*MRI contraindication

*withdrawal/ no response

n= 2

n=10

**Step 3 eligibility assessment: n=42**

One-to-one screening

**Excluded:**

*medication

*other anxiety/ depression

n= 1

**n=8**

n= 7

**Randomisation: n=34**

**Allocation**

**Allocation Waiting Group: n = 17**

Received ‘waiting’: n=17

Withdrawal prior to ‘waiting’: n=0

**Allocation Treatment Group: n = 17**

Received intervention: n=15

Withdrawal prior to intervention: n=2

**Follow-up**

**Discontinued ‘waiting’: n = 3**

**Discontinued intervention: n = 0**

**Analysis**

**Analysed: n = 14**

Excluded: n=0

**Analysed: n = 14**

Excluded due to brain anomaly: n=1

**Supplementary figure 1.** CONSORT flow diagram showing study progress throughout enrolment, allocation, follow-up and analysis. Volunteers who scored at least 4 in an online version of the Panic Disorder Severity Scale – Self-Report (PDSS-SR; (44)) and passed a brief email or phone pre-screening for exclusion criteria were invited to the department for further screening (step 2).
